# Development of a Novel Orthotopic Gastric Cancer Mouse Model

**DOI:** 10.1186/s12575-020-00137-1

**Published:** 2021-01-04

**Authors:** Wonyoung Kang, Leigh Maher, Michael Michaud, Seong-Woo Bae, Seongyeong Kim, Hye Seung Lee, Seock-Ah Im, Han-Kwang Yang, Charles Lee

**Affiliations:** 1 The Jackson Laboratory for Genomic Medicine, 10 Discovery Drive, Farmington, CT 06032 USA; 2 Department of Surgery and Cancer Research Institute, Seoul National University College of Medicine, 103 Daehang-Ro, Jongno-gu, 03080 Seoul, Republic of Korea; 3 Cancer Research Institute, Seoul National University College of Medicine, 103 Daehang-Ro, Jongno-gu, Seoul, 03080 Republic of Korea; 4 Department of Pathology, Seoul National University College of Medicine, 103 Daehang-Ro, Jongno-gu, Seoul, 03080 Republic of Korea

**Keywords:** Gastric cancer, Metastasis, Orthotopic xenograft, Patient-derived xenograft (PDX), Tumor mouse model

## Abstract

**Background:**

Gastric cancer metastasis is a highly fatal disease with a five-year survival rate of less than 5%. One major obstacle in studying gastric cancer metastasis is the lack of faithful models available. The cancer xenograft mouse models are widely used to elucidate the mechanisms of cancer development and progression. Current procedures for creating cancer xenografts include both heterotopic (i.e., subcutaneous) and orthotopic transplantation methods. Compared to the heterotopic model, the orthotopic model has been shown to be the more clinically relevant design as it enables the development of cancer metastasis. Although there are several methods in use to develop the orthotopic gastric cancer model, there is not a model which uses various types of tumor materials, such as soft tissues, semi-liquid tissues, or culture derivatives, due to the technical challenges. Thus, developing the applicable orthotopic model which can utilize various tumor materials is essential.

**Results:**

To overcome the known limitations of the current orthotopic gastric cancer models, such as exposure of tumor fragments to the neighboring organs or only using firm tissues for the orthotopic implantation, we have developed a new method allowing for the complete insertion of soft tissue fragments or homogeneously minced tissues into the stomach submucosa layer of the immunodeficient NOD.Cg-Prkdc^scid^ Il2rg^tm1Wjl^/SzJ (NSG) mouse. With this completely-closed transplantation method, tumors with various types of tissue may be used to establish orthotopic gastric cancer models without the risks of exposure to nearby organs or cell leakage. This surgical procedure was highly reproducible in generating forty-eight mouse models with a surgery success rate of 96% and tumor formation of 93%. Among four orthotopic patient-derived xenograft (PDX) models that we generated in this study, we verified that the occurrence of organotropic metastasis in either the liver or peritoneal cavity was the same as that of the donor patients.

**Conclusion:**

Here we describe a new protocol, step by step, for the establishment of orthotopic xenograft of gastric cancer. This novel technique will be able to increase the use of orthotopic models in broader applications for not only gastric cancer research but also any research related to the stomach microenvironment.

## Background

Gastric cancer is the fourth most common cancer in the world and the second leading cause of cancer-related death worldwide [1–3]. Although chemotherapy has been proven as a standard of care for stage IV gastric cancer [4], the 5-year survival rate of advanced gastric cancer remains at 5–10% [5–7]. Patients with distant metastasis have a particularly poor prognosis, less than 5% survival rate after 5-years, and for these patients, curative surgical resection is no longer an option [6, 8]. Gastric cancer commonly metastasizes to the liver, peritoneum, lymph nodes, lung and bone. The liver is the most common distant metastasis site with a survival rate of 33% at 1-year [9, 10]. The proportion of gastric cancer patients with metastases has grown from 24 to 44% over a 20–year period, showing an urgent need for an optimized approach for both treatment and diagnosis [11]. Due to the high heterogeneity of gastric cancer and the lack of faithful experimental models, the mechanism of gastric cancer metastasis is still largely unknown [12, 13].

**Fig. 1:**
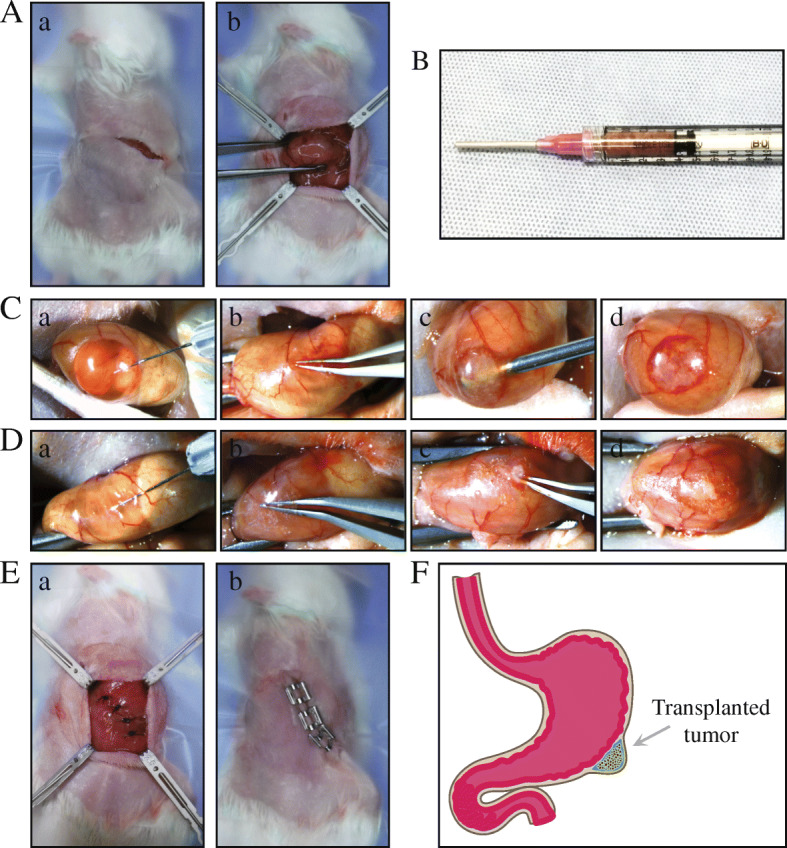
The procedures for the completely-closed orthotopic transplantation of gastric cancer in NSG mice. **A**. **a**. Skin incision made using iris scissors. **b**. Exposing the stomach. Retractors hold both the skin and abdominal walls while forceps gently grab the stomach. **B**. A syringe assembly using an 18 gauge blunt needle and filled with homogenous minced tumor tissue. **C**. **a**. Injection of a low concentration of matrigel. **b**. Widening the entry to the space created under the serosa layer. **c.** Transplantation of up to 20ul of homogenous minced tissue. **d.** Tumor completely loaded into the stomach wall without leakage. **D**. **a**. Injection of a low concentration of matrigel. **b**. Widening the entry to the space created under the serosa layer. **c**. Transplantation of a 3 mm diameter fragment of soft tumor **d**. The tumor tissue completely inserted into the stomach wall without exposure. **E**. **a**. The abdominal wall closure with suture. **b**. The skin closed with wound clips. **F**. An illustration of the properly positioned transplantation site of the completely closed implantation method

Cancer xenograft mouse models are utilized broadly to understand cancer biology and to determine the pharmacological activities of therapeutic agents for therapy development [14–16]. When developing a new mouse model, the transplantation site is the most critical and influential factor affecting tumor initiation, proliferation, and metastasis [17–20]. The heterotopic or ectopic engraftment, when cancer cells or tissues are transplanted into the subcutaneous area of the mouse flank or dorsal regions, is the most commonly used in establishing cell line-derived xenografts (CDXs) or patient-derived xenografts (PDXs) because it is both easy to generate the models and to monitor the tumor volume [21]. However, the heterotopic transplantation site does not provide the crucial microenvironment of the organ where the tumor developed in the patient [22–24]. Indeed, for some tumor types such as colorectal cancer, the subcutaneous model is shown to have a lower engraftment rate than that of the orthotopic model, which provides a better likelihood of tumor settlement in the mouse environment [17, 18]. Orthotopic tumor xenografts are utilized to study metastasis of various tumor types and are proved to be one of the best experimental models to represent the spontaneous metastasis mechanisms [24, 25]. To study gastric cancer metastasis, several orthotopic transplantation methods have been developed. However, each has significant weaknesses which limit its broad use (Table 1); (1) The single-cell suspension injection method is not usable to engraft tissues [26], (2) Tissue transplantation by stitching onto the exposed subserosa of the stomach is a relatively simple method, however, the transplanted tumor tissue is exposed to the peritoneal cavity which can increase the chance of adhesion to neighboring organs. Moreover, only firm tissue fragments can be affixed to the surface with a suture [27], and (3) Tissue transplantation into the subserosa using tissue glue can be achieved by inserting only a firm, ≤ 1mm^3^ tissue fragment through a cut in the serosa then covering the opening with tissue glue to prevent tissue re-emergence and exposure to nearby organs. The exceptionally small size of fragment does not allow recapitulation of heterogeneity of the tumor [28]. There are methods for transplanting tissues orthotopically into the mouse stomach, however, none of them allow for complete insertion into the stomach wall, which is too thin for tissue fragment transplantation. In order to overcome the weaknesses of the current methodologies, we have developed a novel orthotopic transplantation method of gastric cancer, which permits the transplantation of various gastric cancer-derived materials without encountering the problems associated with exposure to neighboring organs or the peritoneal cavity (Fig. 1). Briefly, in the subserosa region of the stomach, a space is created by injecting a low concentrated matrigel solution or saline, the tumor is then transplanted into that space. Following tumor transplantation, the entry of the implantation site is closed thoroughly by compression with a sterile swab. This new orthotopic method, where the stomach tumor forms at the implantation site, most closely resembles tumor development shown in patients. Although advanced surgical techniques may be required, this procedure can be utilized to generate a proper experimental model for multiple research purposes; a model which more accurately mirrors the human patient. In this report, we describe in detail the preparation and surgical procedures of a novel orthotopic transplantation method. We also present the incidence of metastasis and metastatic organ preference shown by four different gastric cancer PDX models which were established using this novel methodology.

**Table 1:** Comparison of orthotopic transplantation methods of stomach tumor

Method	The engraftment volume	Application	Advantage	Limitation
**Cell injection**	< 50 μl	Tumor proliferation and metastasis profile of gastric cancer cell lines. Functional validation of cancer driver genes/pathways by transplanting engineered cell lines. Efficacy test of anti-tumor or anti-metastatic therapeutics.	Minimal organ damage. The fastest transplantation method. High reproducibility.	Possible to inject only single-cell suspension.
**Tissue implantation by suture on the exposed sub-serosa**	Tissue less than 3 mm in diameter	Tumor proliferation and metastasis profile of gastric cancer cell lines and patient-derived tumors.	Possible to transplant patient-derived tissue. Relatively easy procedure as a tissue implantation technic.	Engraft only hard tissue fragment. Tumor exposed to the peripheral cavity. Increase the adhesion to the nearby organs. Difficult to control the tumor heterogeneity.
**Tissue implantation in sub-serosa with glue**	Tissue less than 1 mm in diameter	Tumor proliferation and metastasis profile of gastric cancer cell lines and patient-derived tumors.	Possible to transplant patient-derived tissue.	Engraft only hard 1 mm^3^ tissue fragment. Difficult to control the tumor heterogeneity.
**Completely-closed tissue implantation method**	< 20 μl or Tissue less than 2 mm in diameter	Tumor proliferation and metastasis profile of gastric cancer cell lines and patient-derived tumors. Functional validation of cancer driver genes by using patient-derived organoids system. Possible to test anti-tumor or anti-metastatic therapeutics.	No limit to transplant any types of tissues, including organoids and homogeneous tissue suspension. Tissue insertion completely into the stomach wall.	Technical challenges.

## Results

The present study aims to develop a new method that enables transplantation of various types of materials derived from gastric tumors into the mouse stomach in order to overcome the existing shortcomings of orthotopic methods and also to assess the clinical reliability of the orthotopic gastric cancer model.

### Establishment of the Orthotopic Gastric Cancer PDX Model by Using the Completely-Closed Transplantation Method

The procedures described in the method section are the preparation of a homogenous minced PDX tumor and as well as a protocol for micro-surgery. Tumors from patients who developed gastric cancer liver metastasis or peritoneal metastasis were selected to evaluate the metastatic potential and clinical relevance of the orthotopic model. We chose subcutaneous tumors of four gastric cancer PDX models: SNU-JAX-G364, SNU-JAX-G439, SNU-JAX-G440 and SNU-JAX-G127, which had been established at Seoul National University (SNU) Hospital and Cancer Research Institute, SNU College of Medicine (Seoul, Korea) by collaborating with The Jackson Laboratory for Genomic Medicine (CT, USA). The SNU-JAX-G364 model is a PDX transplanted stomach tumor derived from a patient who developed liver metastasis after primary tumor removal. Both SNU-JAX-G439 and SNU-JAX-G440 are PDXs transplanted liver metastasis that were biopsied from gastric cancer patients. Lastly, the SNU-JAX-G127 model is a PDX transplanted lymph node metastasis derived from a gastric cancer patient who developed peritoneal metastasis. Importantly, traditional subcutaneous methods of these four PDX models have not demonstrated the development of metastatic tumors in mice. The PDX tumors which were passaged twice subcutaneously using NSG mice were prepared for transplantation following Protocol #1 or #2. Depending upon the experimental goals, either cryopreserved or fresh tumors can be transplanted using the proposed methodology. We used PDX tumor freshly harvested from the previous passage in order to reduce any variations between the tumor growth rate in the same group due to the thawing-recovery process.

**Fig. 2:**
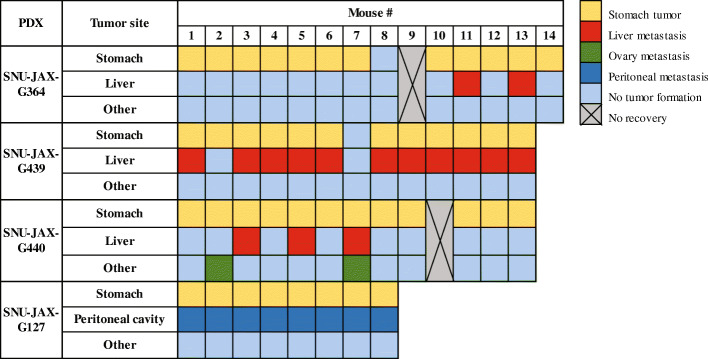
The occurrence of primary and metastatic tumors in gastric cancer orthotopic PDXs. The compiled data of stomach tumor formation (yellow box) determined the transplantation rate of each PDX model. The red or blue box indicates that either liver metastasis or peritoneal seedings occurred in mice, respectively. Green indicates that the mouse developed metastatic tumors at other distant organs than that which occurred in the donor patients, and, in this case, it was the ovary in the SNU-JAX-G440 model

**Fig. 3:**
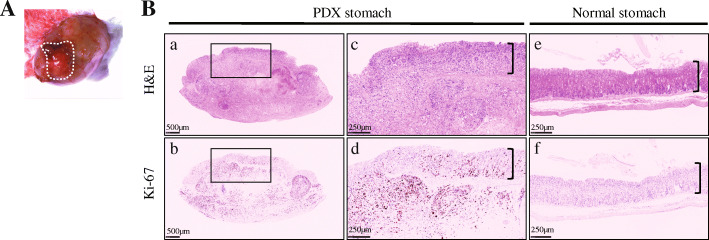
The macro and micro-morphology of the stomach tumor three weeks post transplantation. **A**. The tumor location under the serosa layer of the stomach. **B**. Images of the stomach tumor transection. **a**. A low magnification H&E. **b**. Ki-67 IHC. The regions indicated by the box were magnified to focus on the mucosa and submucosa layers of the stomach. **c**. A high magnification H&E and **d**, Ki-67 IHC. The last two images show a healthy mouse stomach with, **e**. H&E and **f**, Ki-67 IHC. The equal-height bracket indicates the thickness of the mucosa layer in each image

Since the mouse stomach wall thickness is only ~ 0.5 mm, the surgical site was fortified by creating an enlarged space between the serosa and muscle wall by using a 50% mixture of matrigel with RPMI media, the same media used to ship or store patient or PDX tumors. We used the dilute matrigel to enhance tumor settling and maintain the space for ease of transplantation, however, saline may be used as an alternative reagent. In total, 48 orthotopic mice models were generated from the four PDX models (*n* = 8–14 for each model). Post-surgery, two mice did not recover from anesthesia, which gives a surgical success rate of 96%. Tumor formation at the transplantation site was confirmed in forty-four out of forty-six implanted mice, which sets the transplantation success rate at 96% (Fig. 2). From incision to closure, the average time of surgery was 15 min per mouse. At 3 weeks post-surgery, a mouse was sacrificed and the orthotopic transplantation site was observed by macroscopic and microscopic examination to evaluate both the shape and potential leakage of the tumor. We found that the tumor stayed in the subserosa area and no evidence of leakage was seen (Fig. 3A). Also, the vascular formation was actively progressing around the tumor, which could support tumor settlement and proliferation. Histological observation showed that cancer cells that were positive for Ki-67 expression, a proliferative cell marker, invaded into the mucosa layer, which proved the tumor was spreading through stomach layers. Compared to the stomach from a normal NSG mouse, the submucosal region of the PDX stomach was observed as being significantly occupied by the proliferating tumor (Fig. 3B).

### Stomach Tumor Formation and Metastasis in the Orthotopic PDX Models

All mice were monitored twice a week until tumor growth was observed in the stomach. Since we did not utilize an in vivo imaging system, the tumor formation was traced by palpating the abdominal area gently and checking for other physical symptoms (e.i., ascites). When tumor formation was found or suspected, the mice were monitored daily. During the experiments, if mice exhibited any abnormal symptoms, such as weight loss of more than 20%, or gain of more than 20% due to the formation of ascites or tumors, or body condition score (BCS) of equal to or less than 2 [29], they were immediately euthanized. The median experimental days for confirmation of tumor formation in the orthotopic models of SNU-JAX-G364, SNU-JAX-G439, and SNU-JAX-G440 was 58 days (range: 23–153 days), 50 days (range: 35–138 days), and 41 days (range: 34–83 days) post-trans-plantation, respectively. Additionally, the median survival was 124 days (range: 56–183 days), 116 days (range: 66–180 days), and 100 days (range: 70–148 days) post-transplantation in the orthotopic models of SNU-JAX-G364, SNU-JAX-G439, and SNU-JAX-G440, respectively. These PDX models resembled the metastatic patterns shown in the donor patients. The orthotopic models of SNU-JAX-G364, SNU-JAX-G439, and SNU-JAX-G440 developed liver metastasis with incidences of 17, 92, and 25%, respectively. The differences in the metastatic capacity among PDX models may be a result of diversity in the metastatic mechanisms, tumor heterogeneity, and/or the observation time point in our study [30–36]. The liver metastasis in the three donor patients of these PDX models was diagnosed after stomach tumor removal. We euthanized those mice with stomach tumors and then post-mortem observed the metastatic sites. The metastatic capacity may be different among models depending upon the aggressiveness of the stomach tumor and metastasis process at the observation point. The incidence of metastasis may increase if we were able to apply a gastrectomy removing the stomach tumors in mouse models as in the patients’ cases, then this would allow us to further monitor the models until metastasis occurred. The liver was a dominant organ of metastasis in these models, with the exception of ovary metastasis which developed in two out of the twelve mice in the SNU-JAX-G440 group (Fig. 2). Peritoneal metastasis occurred in all of the SNU-JAX-G127 orthotopic model mice.

**Fig. 4:**
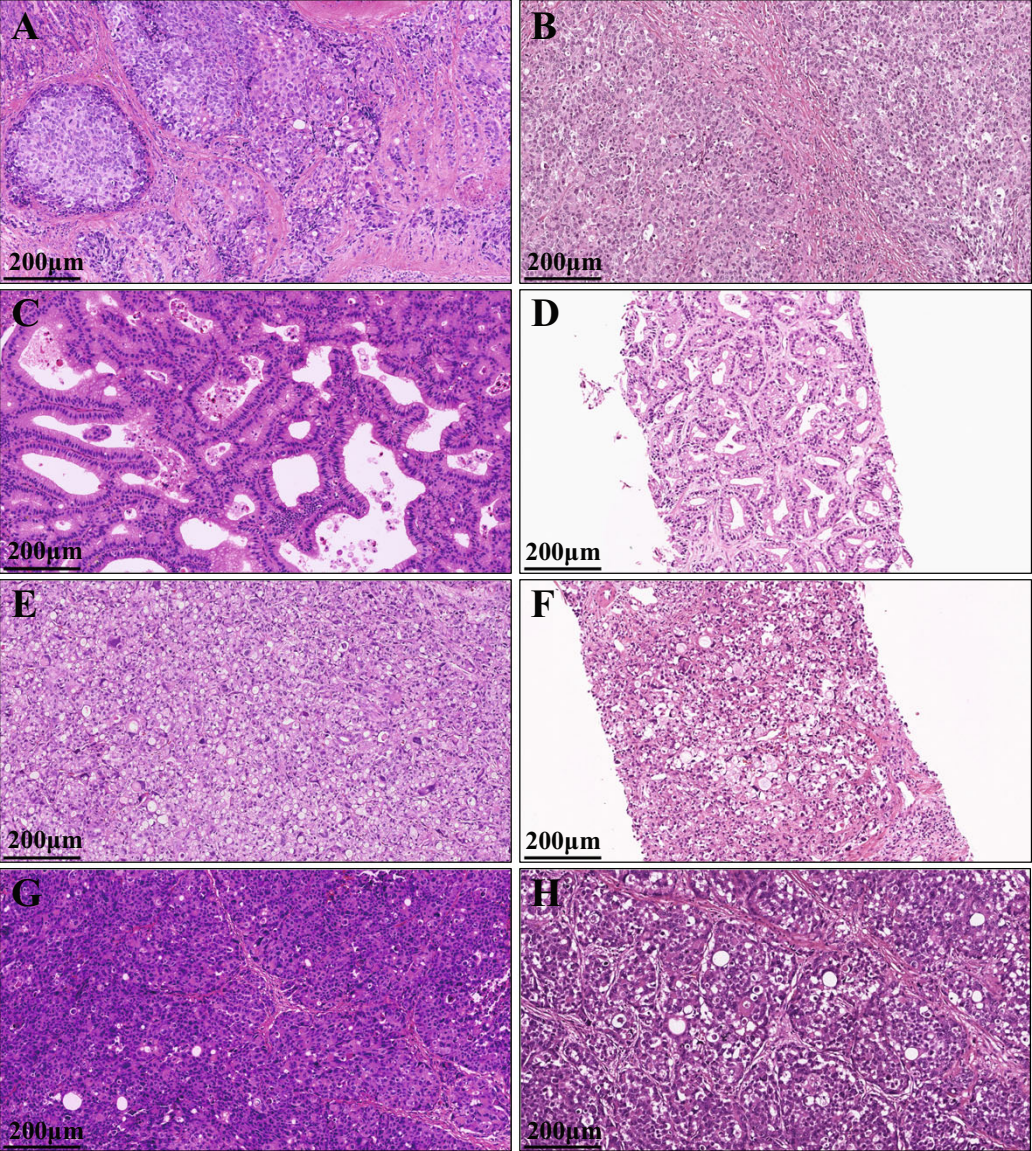
The similarity of tumor pathology between the PDX models and the original patients. The H&E image of **A**. SNU-JAX-G364 PDX tumor, **B**. SNU-JAX-G364 original tumor, **C**. SNU-JAX-439 PDX tumor, **D**. SNU-JAX-439 original tumor, **E**. SNU-JAX-440 PDX tumor, **F**. SNU-JAX-440 original tumor, **G**. SNU-JAX-127 PDX tumor, and **H**. SNU-JAX-127 original tumor (X100, Scale bar = 200 μm

The tumors of these PDX models conserved the identical histological features of donor patients’ original tumors (Fig. 4). The tumor pathology of both the PDXs and the patients was solid type poorly differentiated adenocarcinoma for SNU-JAX-G364 and SNU-JAX-G127, poorly cohesive carcinoma with signet ring cell component for SNU-JAX-G440, and intestinal-type well-differentiated adenocarcinoma for SNU-JAX-G439.

**Fig. 5:**
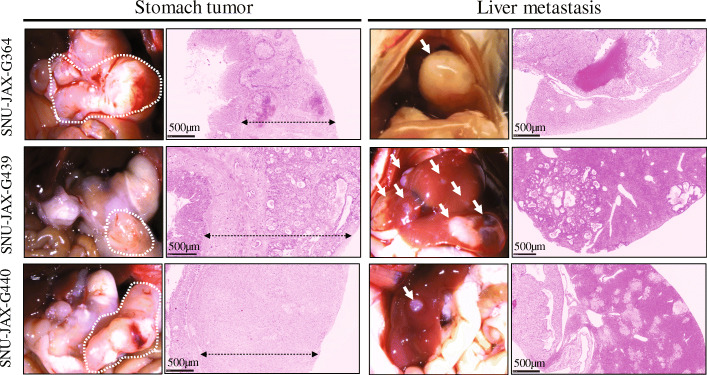
The macro and micro-morphology of the stomach tumor and liver metastasis. The white dotted lines show transplanted cancer cells which grew in each stomach. The black arrow bar on the H&E images of the stomach tumor indicate the region of tumor mass in the submucosa area. Gross examination clearly shows hepatic metastasis that are indicated by white arrows of each model, and H&E images expose micrometastasis

**Fig. 6:**
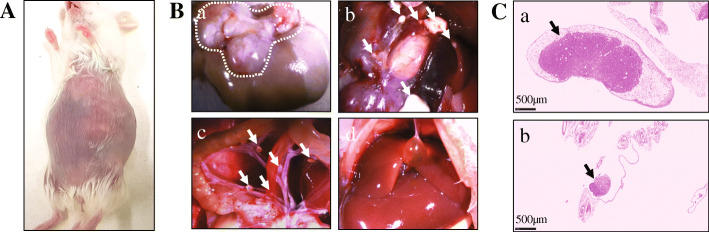
The stomach tumor and peritoneal metastasis in the SNU-JAX-G127 orthotopic model. **A**. The SNU-JAX-G127 orthotopic mouse developed hemorrhagic ascites. The tumor mass was visible and palpated through the abdominal wall. **B**. **a**. The white dotted line outlines where the tumor mass grew in the stomach. **b**, and **c**. Small tumor nodules are shown in the peritoneal cavity and mesenteric areas. **d**. The liver presents without tumor or abnormality. **C**. **a**. The H&E image of tumor modules seeded in the peritoneal cavity and **b**, mesenteric area

All PDX tumor harvests and subsequent implantation into the recipient mouse were performed on the same day; while awaiting surgical implantation all tissues were stored on ice. The orthotopic models were generated sequentially from the first mouse (#1) to the last in each PDX model, and there was no significant difference in the occurrence rate of metastasis among mice due to a variation in tissue transplantation time (Fig. 2). Stomach tumors were large enough to attach to the liver, however, there was no direct invasion of the tumor into the liver parenchyma. The liver metastatic tumors were found in lobes distant from the stomach and were harvested to investigate the histological morphology. Each model exhibited different features of metastatic patterns. The SNU-JAX-G364 liver metastatic tumor was a large mass in the liver which overwhelmed the hepatocytes and normal hepatic structures. The SNU-JAX-G439 orthotopic model showed the highest incidence of extremely invasive liver metastasis. The livers with metastatic tumors were fragile and had lesions of various colors, such as yellow, dark green, black, and white, suggesting that there might be severe damage to the normal function of the liver. In the gross examination, the SNU-JAX-G440 mice had only small tumor masses in the liver without any other abnormality. However, many micro-metastatic tumors were observed in the H&E images (Fig. 5). The SNU-JAX-G127 orthotopic model developed hemorrhagic ascites along with cancer cell seeding in mesenteric sites and organs, including kidney, intestines, or ovary in the peritoneal cavity (Fig. 6). The median experimental day of tumor formation confirmation was 39 days (range: 35–41 days) post-transplantation and the median survival was 72 days (range: 58–106 days) post-transplantation in the orthotopic models of SNU-JAX-G127.

## Discussion

Despite the critical need to find the optimal strategy for diagnosis and treatment of gastric cancer metastasis, the paucity of reliable translational animal models is a huge impediment to investigating this disease. We have established a novel method which enables the transplantation of a multitude of tissue types into the stomach orthotopically. Both the advantages and limitations of this new approach, a completely-closed implantation method, were compared with other commonly used methods in Table 1. There are several crucial points to consider to conduct a successful procedure. Firstly, in order to prevent severe dehydration or hypothermia and ensure the timely recovery of the mouse, throughout surgery, the organs must be kept hydrated by covering with wet gauze or supplying warm sterile saline, and the body temperature must be maintained by using an appropriate warming pad. Secondly, the duration of surgery is a factor which influences the achievable scale of an experiment, as well as the success of each surgery. In order to minimize the critical surgical time, it is important to work as a team with staff both monitoring mouse recovery and supporting the surgery. Thirdly, the most critical and challenging step in this model is creating a space by injection of solution just below the serosa. It requires extensive practice to ensure both correct placement and repeatability. If injection of the solution is too deep into the muscular layer or submucosal layer, pillar structures of connective tissue may form inside the space and obstruct transplantation of tissue. Fourthly, adding the matrigel to the tumor materials may be beneficial to complete this procedure. Matrigel is a well-known gelatinous protein mixture consisting of extracellular matrix substrates and is used to increase engraftment rate in PDX models [37]. In our study, matrigel might support the stabilization of the homogenous minced tumors in the transplantation site by its characteristic transformation into a gel when responding to body temperature. This transformation provides a less fluidic-matrix which may support the prevention of leakage of tumors from the injection site during the procedure, helping to accomplish complete closure of the transplantation site. Lastly, complete closure of the transplantation site is essential to prevent the leakage which causes adhesion to neighboring organs or to the incision on the abdominal wall. Although we found that with slight pressure from a swab on to the injection site, complete-closure was achieved, as a supplementary material, a tissue adhesive can be applied to seal the site completely, and the gelfoam, also, can be used to cover the entry of the transplantation area. In this study, we observed stomach tumor and metastasis in the orthotopic PDX models after the animal was sacrificed, but with the use of imaging equipment, such as small animal magnetic resonance imaging (MRI) or ultrasound, we will be able to investigate the complex cascades of gastric cancer metastasis, created by applying various types of tumor materials, in living animals. Furthermore, the genes responsible for the liver metastasis of gastric cancer will be further studied and functionally validated by utilizing the metastatic tumors and the primary stomach tumors from each PDX model we have developed in this study.

## Conclusions

Existing orthotopic implantation methods of the stomach tumor are used to generate the orthotopic gastric cancer models to study cancer biology and/or metastasis. However, due to technical difficulties, only specific types of material have been able to be transplanted, such as single-cell suspension or a firm fragment of tumor [26–28]. Our study reports a novel surgical procedure that is able to overcome the weaknesses of the existing methodologies. Although this new method may require a higher level of surgical skill than the others, it will generate a reliable and consistent orthotopic model that transplants an equal amount of either homogeneously minced tissue or other soluble tissue materials. Potentially, this novel completely-closed transplantation method can be used for investigating the functions of biomarkers, testing the efficacy of antitumor or anti-metastatic drugs, and be utilized for more broad applications such as studies employing gastric cancer organoids, or non-cancerous materials that relate to the stomach microenvironment.

## Methods

### Protocol #1. Preparation of Fresh Homogeneous Minced Tumor

1. All procedures are performed in the biosafety cabinet.
2. The tumor tissue is washed with cold phosphate-buffered saline (PBS) and placed in a petri-dish, which is sitting on an icepack. It is then minced using surgical forceps and sterile single edge razor blades into tiny fragments that are less than 1 mm diameter.
3. During the preparation of minced tumor materials, a 50% matrigel solution diluted with RPMI 1640 media is added to the tumor matrix to avoid drying of the tissues. As a tumor contains some amount of the fluids in it, the supplementary solution volume can be adjusted considering homogenous of the tumor matrix. In this study, we applied the diluted matrigel about 10–20% of tumor volume.
4. To prevent bubble formation in the minced tissues, the tumor matrix is loaded through the plunger end of a 5 ml syringe using surgical forceps. Any air is forced out after applying pressure with the plunger. Using this 5 ml syringe, the bubble-free tumor matrix is introduced into a disposable 1 ml syringe, which is then assembled with an 18 gauge needle and stored on ice until use (Fig. 1B).

### Protocol #2. Preparation of Soft Tumor Fragment

1. All procedures are performed in the biosafety cabinet.
2. After washing with cold PBS, the tissue fragment is placed in a Petri dish sitting on an icepack. Using surgical forceps and sterile single edge razor blades, the tissue is divided into small fragments (3X3X3 mm^3^).
3. An optimal soft tumor fragment is selected for transplant and is placed in 200ul, 50% matrigel solution diluted with RPMI 1640 media and stored on ice until use.

### Protocol #3. Creation of a Void in the Stomach Wall

**Fig. 7:**
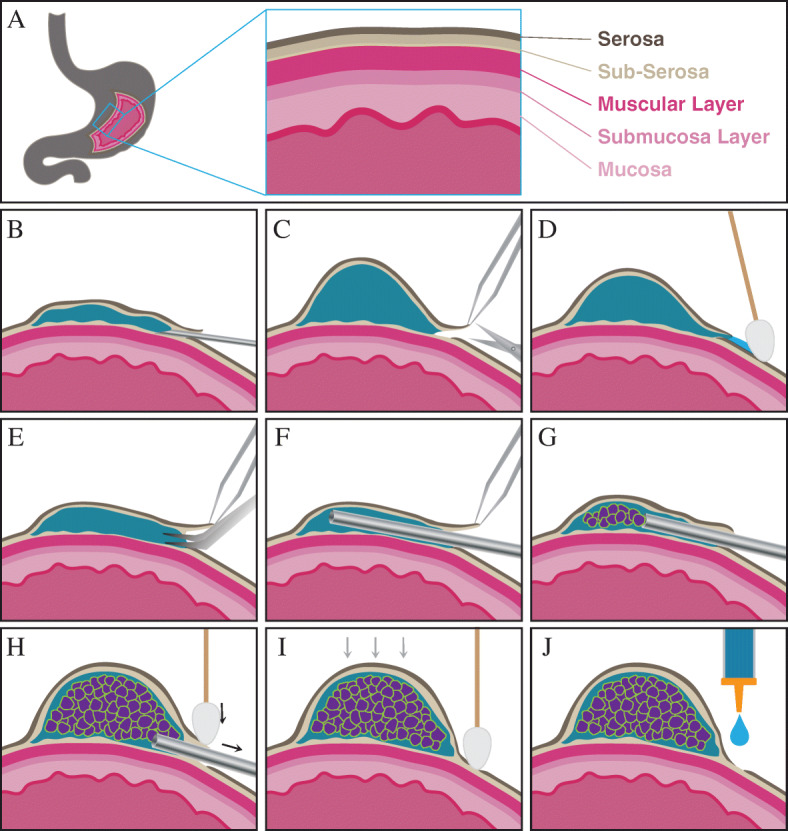
The procedure to transplant the homogeneous tissues under the stomach serosa layer. **A**. The layers compose the stomach wall. **B**. Using a 31 gauge needle on an insulin syringe 30–40 ul of either 50% matrigel diluted with RPMI media or sterile saline is injected into the subserosa area. **C**. The serosa over the injection site is incised 2–3 mm with Vannas Iridocapsulotomy Scissors in order to generate the entrance to insert the 18 gauge need and evacuate the space-creating 50% matrigel or saline. **D**. The solution which had leaked is wiped with sterile gauze or cotton swabs. **E**. A cutting site is widened using Micro-Blunted Tip Dumont Forceps or Moria Iris Forceps. **F**. An 18 gauge needle is placed into the inner-space. **G**. Fill the tissue slowly from the opposite area of the entrance. **H**. After implantation, the entry site is pressed lightly with a sterile cotton swab and the needle is gently removed. **I**. Giving an extremely light and gentle pressure the transplantation site to confirm the leaking and the entry site is pressed with a sterile cotton swab for 20–30 s, on average, until it closes completely. **J**. As an auxiliary closure, tissue adhesive is beneficial to cover the incision site of the stomach serosa

1. All procedures are performed in the biosafety cabinet in the Biosafety Label 2 (BSL2) procedure room of the animal facility at UConn Health. All work follows the animal protocols approved by both the Institutional Animal Care and Use Committee (IACUC) of UConn Health and The Jackson Laboratory (#101660–0920 and #17065, respectively).
2. The bodyweight of an 8 weeks-old, female NSG mouse is measured and then is anesthetized by intraperitoneal (IP) injection of 100 mg/kg ketamine mixed with 10 mg/kg xylazine. An analgesic is also given IP pre-surgery (Ketoprofen, 5 mg/kg) and all are administered using disposable 1 ml syringes.
3. The mouse is placed on the mouse warming pad for pre-operative preparation.
4. The fur of the region from the ventral abdomen and the left flank, in an area bounded to the mid thorax and pelvis, is removed using clippers. The fur removal area can be extended to the dorsal midline to remove the fur of the left dorsal region.
5. Loose fur is removed with adhesive tape, dry gauze or gauze slightly dampened with ethanol.
6. Ophthalmic ointment is placed on the eyes to prevent drying of the cornea.
7. The skin is disinfected with both 70% ethanol and chlorhexidine using sterile swabs. The application of 70% ethanol starts in the center of the proposed incision site and works outward in ever-widening circles to cover the entire clipped area, and then using a new sterile swab, repeat again. Chlorhexidine is applied in the same manner.
8. To keep the body temperature during the surgery, a mouse warming pad, of which size fits for the microscopic surgery, is placed under the surgical microscope and covered by a sterile disposable drape.
9. The mouse is placed in a dorsal recumbent position under the surgical microscope and a sterile Invisishield Aperture Surgical Drape is positioned over the surgical site.
10. Using either Von Graefe Iris Forceps or Extra Fine Graefe Forceps and Iris Scissors, a 2.0 cm skin incision is made immediately caudal and parallel to the caudal most rib (Fig. 1Aa).
11. The underlying muscle is incised and held by retractors exposing the stomach (Fig. 1Ab) using a fresh set of Von Graefe Iris Forceps or Extra Fine Graefe Forceps and Iris Scissors.
12. Ring Forceps are used to hold the stomach in place during the surgery. A moistened gauze can be used to position the stomach exposing the greater curvature of the glandular portion of the stomach.
13. For each animal one 31 gauge insulin syringe containing dilute matrigel of sterile saline is prepared and stored on ice until used.
14. In order to prepare a space for tissue implantation, using a 31 gauge needle on an insulin syringe 30–40 ul of either 50% matrigel diluted with RPMI media or sterile saline is injected into the subserosa area of the stomach (Fig. 1Ca and Fig. 7B).
15. The serosa over the injection site is incised 2–3 mm with Vannas Iridocapsulotomy Scissors in order to evacuate all of the space-creating 50% matrigel or saline. (Fig. 1Cb and Fig. 7C). The matrigel or saline which had leaked is wiped with sterile gauze or cotton swabs (Fig. 7D).

### *Protocol #4.* Transplantation of Homogenous Minced Tumor

1. Using Micro-Blunted Tip Dumont Forceps or Moria Iris Forceps (Fig. 7E), a cutting site is widened in order to place an 18 gauge needle into the inner-space (Fig. 7F) and inject up to 20 ul of tissue (Fig. 1Cc and Fig. 7G).
2. After slow implantation, the needle is gently removed (Fig. 7H) and the entry site is pressed lightly with a sterile cotton swab for 20–30 s until it closes completely. Giving an extremely light and gentle pressure the transplantation site might help to confirm whether there is anything leaking from it. (Fig. 1Cd and Fig. 7I).
3. As an auxiliary closure, tissue adhesive or gelfoam can be used to cover the incision site of the stomach serosa (Fig. 7J).

### *Protocol #5.* Transplantation of a Soft Tumor Fragment

1. Using Micro-Blunted Tip Dumont Forceps or Moria Iris Forceps the transplant site is opened gently (Fig. 1Db).
2. Dumont Forceps are used to insert the edge of the tissue fragment into the incision site (Fig. 1Dc).
3. The tissue is gently pushed into the pocket until completely inserted and then by nudging it through the surface of the serosa, it is positioned at the far end of the space opposite to the entry (Fig. 1Dd).
4. The incision site is closed by applying gentle pressure with a sterile cotton swab.

### Protocol #6. Closure of the Surgical Site And Animal Recovery

1. After observing there is no leakage, the stomach is gently advanced away from the opening.
2. The incision in the abdominal wall is closed with a 5–0 or 6–0 absorbable suture with a swaged on the needle using Micro Needle Holders (Fig. 1Ea).
3. 0.1% bupivacaine is applied to edges of the skin incision.
4. The skin incision is closed with wound clips (Fig. 1Eb).
5. After surgery, the mouse is placed in lateral recumbency in a clean, and warm cage.
6. The body weight of the mouse is measured and recorded
7. Using a disposable 5 ml syringe, sterile saline warmed by the heating pad (temperature: 35–37 °C) is given by subcutaneous injection for hydration (1 ml per 25 g bodyweight).
8. The recovery cage is placed on a cage heating pad to be warmed to 30–37 °C, and the mouse is provided a means to move away from the heat source once awake.
9. The mouse is maintained in the individually ventilated cages (IVC) once it is able to walk around the cage.

### Protocol #7. Microscopic Morphology Observation of Tumors

1. The mice were euthanized by 5% carbon dioxide once it was observed that the bodyweight either decreased or increased by more than 20% or if abnormal physical symptoms occurred, such as ascites or decline in motility.
2. Both stomach and metastatic tumors were detected using a surgical microscope and pictures were taken.
3. The tumor masses were collected and, using a stainless steel tissue slicer matrix, a 1 mm thick slice was generated for pathology procedures.
4. The slice was fixed with 10% neutral formalin for 24 h at 4 C°.
5. Tissue was embedded in paraffin, and Hematoxylin & Eosin (H&E) staining was performed according to standard protocols.
6. For immunohistochemistry (IHC), both the normal stomach and the tumor were collected at 3 weeks post-surgery then stained using the Ki-67 antibody.
7. The slide images were scanned using the Hamamatsu NanoZoomer HT2.0 scanner, and using NDPIView2 software, the microscopic morphology was examined.

### Materials

Fresh or cryopreserved gastric cancer patient or xenograft tumor tissue.

Petri dish 100 mm (Corning, 353,003).

Matrigel™ Basement Membrane Matrix (Corning, 354,234).

RPMI 1640 media (Gibco, 11,875,119).

18 gauge blunt needle (Weller Needles, KDS181P).

Sterile cotton swabs (Med Vet International, CTA6STRLX10).

Invisishield Aperture Surgical Drape (Medline, DYNJSD1020).

Sterile disposable drape (Medline, NON21001).

Disposable 1 ml syringe (Medline, B-D309628Z).

Disposable 5 ml syringe (Medline, B-D309646Z).

Disposable 31 gauge insulin syringe (BD, SY8290328289).

Gelfoam (Medline, 0009–0396-05).

Tissue adhesive (B. Braun, Histoacryl).

Absorbable suture (Medline, ETHJ385H).

Autoclip kit and wound clips (Fine Science Tools, 12,020–00).

50 ml falcon tube (Corning 352,098).

Mouse warmer (Stoelting, 53,850 M).

Cage heating pads (Stoelting, 53850C).

Sterile gauze.

Scale.

Sterile saline.

Clipper.

Digital camera.

Icepacks.

Ice

### Animal

Eight weeks-old, female NOD.Cg-Prkdc^scid^ Il2rg^tm1Wj^l/SzJ (NSG) mouse (The Jackson Laboratory, #005557).

### Surgical Instruments

**Tissue preparation kit:**

Dumont Forceps (Fine Science Tools, 11,251–35).

Single Edge Razor Blades (Electron Microscopy, 71,970-WA).

**Microsurgical kit:**

Ring Forceps (Fine Science Tools, 11,103–09).

Dumont Forceps (Fine Science Tools, 11,251–35).

Micro-Blunted Tip Dumont Forceps (Fine Science Tools, 11,253–25).

Moria Iris Forceps (Fine Science Tools, 11,373–12).

Extra Fine Graefe Forceps (Medline, MDS0757651).

Von Graefe Iris Forceps (Fine Science Tools, 11,051–10).

Iris scissors (Medline, MDS0805210).

Micro Needle Holders (Medline, MDS2442014).

Vannas Iridocapsulotomy Scissors (Fine Science Tools, 91,500–09).

**Mouse retractor set (Fine Science Tools, 18,200–20).**

**Dissection kits:**

Von Graefe Iris Forceps (Fine Science Tools, 11,051–10),

Dumont Forceps (Fine Science Tools, 11,251–35).

Iris scissors (Medline, MDS0805210).

Single Edge Razor Blades (Electron Microscopy, 71,970-WA).

Stainless steel tissue slicer matrix (Zivic, HSRS005–1).

### Reagents

Topical anesthesia: bupivacaine HCl (0.1%) (Hospira, Inc., NDC 0409–1159-18).

Puralube® Ophthalmic Ointment (Dechra Vet, NDC 17033–211-38).

70% ethanol (Fisher scientific, NC9663244).

Chlorhexidine (Durvet Inc., NDC 30798–668-35).

Phosphate-buffered saline (Gibco, 20,012,027).

10% neutral formalin (Fisher scientific, SF98–4).

Hematoxylin (Thermo scientific, 72,804).

Eosin (Leica, 3,801,600).

Ki-67 Antibody (Thermo Scientific, RM-9106).

### Drug Concentration

**Concentration of anesthetics** 10 mg/ml Ketamine (Pfizer, 00409205105) plus 2 mg/ml Xylazine (Akorn, 59,399–110-20) diluted with sterile saline.

**Concentration of analgesics** 1 mg/ml Ketoprofen (Zoetis, 10,004,031) diluted with sterile saline.

### Equipment

Surgical microscope assembled with the camera (Leica, M156C).

BSL2 Biosafety cabinet.

IVC and cage racks.

Carbone dioxide rodent euthanasia system.

NanoZoomer slide scanner (Hamamatsu, HT2.0).

## Data Availability

Not applicable.

## Abbreviations

NSG: Immunodeficient NOD.Cg-Prkdc^scid^ Il2rg^tm1Wjl^/SzJ
PDX: Patient-derived xenograft
CDXs: Cell line-derived xenografts
BCS: Body condition score
MRI: Magnetic resonance imaging
PBS: Phosphate-buffered saline
BSL2: Biosafety Label 2
IACUC: Institutional Animal Care and Use Committee
IP: Intraperitoneal
IVC: Individually ventilated cages
H&E: Hematoxylin & Eosin
IHC: Immunohistochemistry
