# Correction to: Development of a Novel Orthotopic Gastric Cancer Mouse Model

**DOI:** 10.1186/s12575-021-00146-8

**Published:** 2021-04-07

**Authors:** Wonyoung Kang, Leigh Maher, Michael Michaud, Seong-Woo Bae, Seongyeong Kim, Hye Seung Lee, Seock-Ah Im, Han-Kwang Yang, Charles Lee

**Affiliations:** 1 The Jackson Laboratory for Genomic Medicine, 10 Discovery Drive, Farmington, CT 06032 USA; 2 Department of Surgery and Cancer Research Institute, Seoul National University College of Medicine, 103 Daehang-Ro, Jongno-gu, 03080 Seoul, Republic of Korea; 3 Cancer Research Institute, Seoul National University College of Medicine, 103 Daehang-Ro, Jongno-gu, Seoul, 03080 Republic of Korea; 4 Department of Pathology, Seoul National University College of Medicine, 103 Daehang-Ro, Jongno-gu, Seoul, 03080 Republic of Korea

**Correction to: Biol Proced Online 23, 1 (2021)**

10.1186/s12575-020-00137-1

Following publication of the original article [1], the below Acknowledgement was missing.

**Acknowledgements**

The authors would like to thank Jane Winnie Cha, a senior designer of The Jackson Laboratory, for her efforts and technical assistance in creating the illustrations. Also, we gratefully acknowledge the contribution of the Histology Service at The Jackson Laboratory for expert assistance with the work described in this publication.

The original article [1] has been corrected.
